# Distribution, dynamic evolution, and clinical outcomes of patients with advanced breast cancer according to HER2 expression

**DOI:** 10.1186/s12885-023-10634-7

**Published:** 2023-02-21

**Authors:** Qin Shi, Jing Yu, Deyue Liu, Fang Ren, Jiayi Wu, Kunwei Shen

**Affiliations:** 1 Department of Breast and Thyroid Surgery, Hangzhou Linping District Maternal and child Care Hospital, Hangzhou, China; 2 Department of General Surgery, Comprehensive Breast Health Center, Ruijin Hospital, Shanghai Jiao Tong University School of Medicine, 197 Ruijin Er Road, 200025 Shanghai, China; 3 Department of Breast and Thyroid Surgery, Shanghai General Hospital, Shanghai JiaoTong University School of Medicine, Shanghai, China

**Keywords:** Breast cancer, Disease recurrence, HER2-low, Clinical outcome, Evolution

## Abstract

**Background:**

Novel antibody‒drug conjugates (ADC) have shown great efficacy in HER2-low advanced breast cancer. However, the clinical features of HER2-low disease still need to be clarified. The current study aims to evaluate the distribution and dynamic change in HER2 expression in patients with disease recurrence and the clinical outcome of those patients.

**Methods:**

Patients with pathologically diagnosed relapsed breast cancer between 2009 and 2018 were included. Samples were considered HER2-zero when the immunohistochemistry (IHC) score was 0, HER2-low when the IHC score was 1 + or 2 + with negative fluorescence in situ hybridization (FISH) results, and HER2-positive when the IHC score was 3 + or the FISH results were positive. Breast cancer-specific survival (BCSS) was compared among the three HER2 groups. Changes in HER2 status were also evaluated.

**Results:**

A total of 247 patients were included. Among recurrent tumors, 53 (21.5%) were HER2-zero, 127 (51.4%) were HER2-low, and 67 (27.1%) were HER2-positive. The HER2-low subtype represented 68.1% of the HR-positive breast cancer group and 31.3% of the HR-negative group (P < 0.001). This three-group classification of HER2 status was prognostic in advanced breast cancer (P = 0.0011), with HER2-positive patients having the best clinical outcome after disease recurrence (P = 0.024), while only marginal survival advantages were observed in HER2-low patients versus HER2-zero patients (P = 0.051). In the subgroup analysis, the survival difference was observed only in patients with HR-negative recurrent tumors (P = 0.0006) or with distant metastasis (P = 0.0037). The overall discordance rate of HER2 status between primary and recurrent tumors was 38.1%, with 25 (49.0%) primary HER2-zero patients and 19 (26.8%) HER2-positive patients shifting to HER2-low at recurrence.

**Conclusion:**

Nearly half of the advanced breast cancer patients had HER2-low disease, which indicates a poorer prognosis than HER2-positive disease and marginally better outcomes than HER2-zero disease. During disease progression, one-fifth of tumors convert to HER2-low entities, and the corresponding patients may benefit from ADC treatment.

**Supplementary Information:**

The online version contains supplementary material available at 10.1186/s12885-023-10634-7.

## Introduction

Human epidermal growth factor receptor 2 (HER2), encoded by ERBB2, serves as an important prognostic biomarker as well as a therapeutic target in breast cancer [1–3]. The HER2-positive subtype only represents 15–20% of all breast cancers [3, 4]. The majority of breast tumors are HER2-negative, defined as an immunohistochemistry (IHC) score of 0, 1+, or 2 + with a negative in situ hybridization (ISH) result according to the American Society of Clinical Oncology/College of American Pathologists (ASCO/CAP) guidelines. Recently, a new classification termed “HER2-low” breast cancer has emerged [5].

Based on the HER2 testing algorithm, HER2-low breast cancer includes tumors with low (IHC 1+) or moderate (IHC 2 + with negative ISH) HER2 expression [5]. This type accounts for 45–55% of all cases [5] and exhibits different biological features from the HER2 completely negative (IHC 0) subtype [6–8]. HER2-positive breast cancer is an aggressive phenotype with high recurrence rates and inferior survival outcomes. However, ambiguous data support that HER2-low status has unique prognostic or predictive value [7]. In early-stage breast cancer, the NSABP B-47 trial demonstrated that those patients failed to gain benefit from trastuzumab in addition to adjuvant chemotherapy [9]. Regarding their long-term clinical outcomes, no unanimous conclusion can be drawn [6, 8, 10, 11]. On the other hand, in the advanced setting, survival data for HER2-low patients are still lacking. In clinical practice, the HER2-low subtype shares the same treatment strategy with luminal-like or triple-negative breast cancer.

However, the low level of HER2 expression could still provide a potential therapeutic target for this entity in the metastatic setting. In laboratory research, a novel antibody-drug conjugate (ADC) showed promising antitumor activity in HER2-low breast carcinoma [12–14]. In subsequent phase I clinical trials, ADCs resulted in an objective response rate (ORR) of 30–40% in HER2-low advanced breast cancer patients, possibly due to the bystander killing effect [15, 16]. As a consequence, phase III clinical studies are being conducted to compare the efficacy of HER2-targeting ADCs with chemotherapy in this newly defined disease subtype [17, 18], which had revealed a new opportunity for anti-HER2 treatment^34^.

To date, limited research has focused on the clinical outcome of HER2-low recurrent breast cancer. Discordance in HER2 status between the tumor at first diagnosis and recurrence has rarely been reported since HER2-low was considered as a subtype. The current study aimed to evaluate the distribution of HER2 expression in recurrent breast cancer, to assess the survival data of those patients, and to illustrate the evolution of HER2 status during disease progression.

## Materials and methods

### Study population

Breast cancer patients who received surgical treatment at Ruijin Hospital from Jan. 2009 to Dec. 2018 and had confirmed disease recurrence were retrospectively reviewed. Those undergoing radiology-guided biopsy or resection of the recurrent lesion were included. The exclusion criteria were as follows: (1) recurrent tumors diagnosed as pure ductal carcinoma in situ (DCIS) or mesenchymal tumors; (2) unknown IHC results for tumors at first diagnosis and recurrence; and (3) unknown fluorescence in situ hybridization (FISH) results for HER2 if the IHC result was 2+. Data on clinicopathological features, treatment schedule, and disease outcome were retrieved from the Shanghai Jiao Tong University Breast Cancer Database (SJTU-BCDB).

### Outcome endpoint

Standardized Definitions for Efficacy End Points (STEEP) version 2.0 were used as a reference when selecting the outcome endpoint [19]. Breast cancer-specific survival (BCSS) was calculated from the date of the first recurrence until the date of death from breast cancer. Locoregional recurrence (LRR) included ipsilateral invasive tumor recurrence in the breast or chest wall and ipsilateral node recurrence in the axilla, supraclavicular, or internal mammary regions. Distant metastasis (DM) included bone metastasis, visceral metastasis, and soft tissue metastasis. Contralateral breast cancer (CBC) only refers to invasive tumors. For patients with two or more sites of disease recurrence, the type of the first site of recurrence will be recorded.

### Pathological assessment, IHC, and FISH

The pathological evaluation was performed at the pathological department of Ruijin Hospital by at least two experienced pathologists. IHC analysis was conducted on 4 μm formalin-fixed, paraffin-embedded tissue sections with the procedure detailed in our previous study [20]. Positivity of hormone receptor (HR), including ER and PR, was defined as ≥ 1% of tumor cells having nuclear staining.

Evaluation of HER2 expression referred to the latest version of ASCO/CAP guidelines at the time of diagnosis, which had been released in 2007 and updated in 2013 and 2018 [4, 21, 22]. For tumors with HER2 IHC 2+, dual-probe FISH tests were further performed on the same specimen by using the PathVysion HER2 DNA FISH Kit (Vysis Inc, Downers Grove, IL). HER2-positive referred to an IHC score 3 + or an IHC score of 2 + with a positive FISH result. HER2-negative tumors were further classified according to IHC and FISH results. HER2-zero referred to an IHC score of 0. HER2-low referred to an IHC score of 1 + or an IHC score of 2 + with negative FISH.

### Statistical analysis

Clinicopathological characteristics were compared among the HER2-zero, HER2-low, and HER2-positive groups by the chi-square test or Fisher’s exact test if necessary. The distribution of continuous variables was compared using Kruskal‒Wallis test. Survival outcomes were compared among groups by using Kaplan‒Meier curves with the log-rank test. Cox regression models were applied to calculate the hazard ratio with a 95% confidence interval (CI). Statistical analyses were conducted using R software (version 4.0.5). A two-sided P value < 0.05 was considered to indicate statistical significance.

## Results

### Clinicopathological characteristics

A total of 247 patients were included (Figure S1), and the baseline characteristics of recurrent tumors are shown in Table 1. The median time between the primary diagnosis and the biopsy or resection of the recurrent lesion was 30.5 months. The median age at recurrence was 54 (range 26 to 93) years, with 157 patients (63.6%) older than 50 years at the time of recurrence. Among the 254 recurrence site samples, 89 (36.0%) were LRR, 92 (37.2%) were distant metastasis, and 66 (26.7%) were CBC samples. A total of 31 patients (12.6%) had disease recurrence within 1 year after the first diagnosis, 68 patients (27.5%) had recurrence between 1 and 2 years, and 148 (59.9%) patients relapsed beyond 2 years. HR-positive tumors accounted for 54.7% (135) of all tumors. The median staining rate of Ki-67 remained 30% in the recurrent tumors, and 38.1% (94) of the tumors had Ki-67 > 30%.

**Table 1:** Patients’ characteristics according to HER2 status of the relapsed site

	Total n (%)	HER2-zero n (%)	HER2-low n (%)	HER2-positive n (%)	P-value
**Age at recurrence (Median, range)**					0.268
	54 (26–93)	54 (30–93)	54 (26–88)	52 (28–77)	
**Age at recurrence (years)**					0.227
≤ 50	90	19 (21.1)	41 (45.6)	30 (33.3)	
> 50	157	34 (21.7)	86 (54.8)	37 (23.6)	
**BCFI (months)**					0.217
< 12	31	5 (16.1)	14 (45.2)	12 (38.7)	
12–24	68	20 (29.4)	31 (45.6)	17 (25.0)	
> 24	148	28 (18.9)	82 (55.4)	38 (25.7)	
**Type of the first relapse**					0.703
LRR	89	19 (21.3)	48 (53.9)	22 (24.7)	
DM	92	22 (23.9)	42 (45.7)	28 (30.4)	
CBC	66	12 (18.2)	37 (56.1)	17 (25.8)	
**Site of biopsy or resection**					0.973
Ipsilateral breast	34	8 (23.5)	17 (50.0)	9 (26.5)	
Chest wall	35	8 (22.9)	20 (57.1)	7 (20.0)	
Lymph node	20	3 (15.0)	11 (55.0)	6 (30.0)	
Contralateral breast	66	12 (18.2)	37 (56.1)	17 (25.8)	
Lung	21	6 (28.6)	11 (52.4)	4 (19.0)	
Liver	26	6 (23.1)	11 (42.3)	9 (34.6)	
Bone	18	4 (22.2)	9 (50.0)	5 (27.8)	
Others	27	6 (22.2)	11 (40.7)	10 (37.0)	
**HR status of relapse site**					< 0.001
Positive	135	23 (17.0)	92 (68.1)	20 (14.8)	
Negative	112	30 (26.8)	35 (31.3)	47 (42.0)	
**Ki-67 index of relapsed site@**					0.267
≤ 30%	124	22 (17.7)	69 (55.6)	33 (26.6)	
> 30%	94	24 (25.5)	43 (45.7)	27 (28.7)	

@ 29 patients had unknown Ki-67 index for relapsed site

Abbreviation: BCFI = Breast cancer-free interval, LRR = local-regional recurrence, DM = distant metastasis, CBC = contralateral breast cancer, HR = hormone receptor

**Fig. 1:**
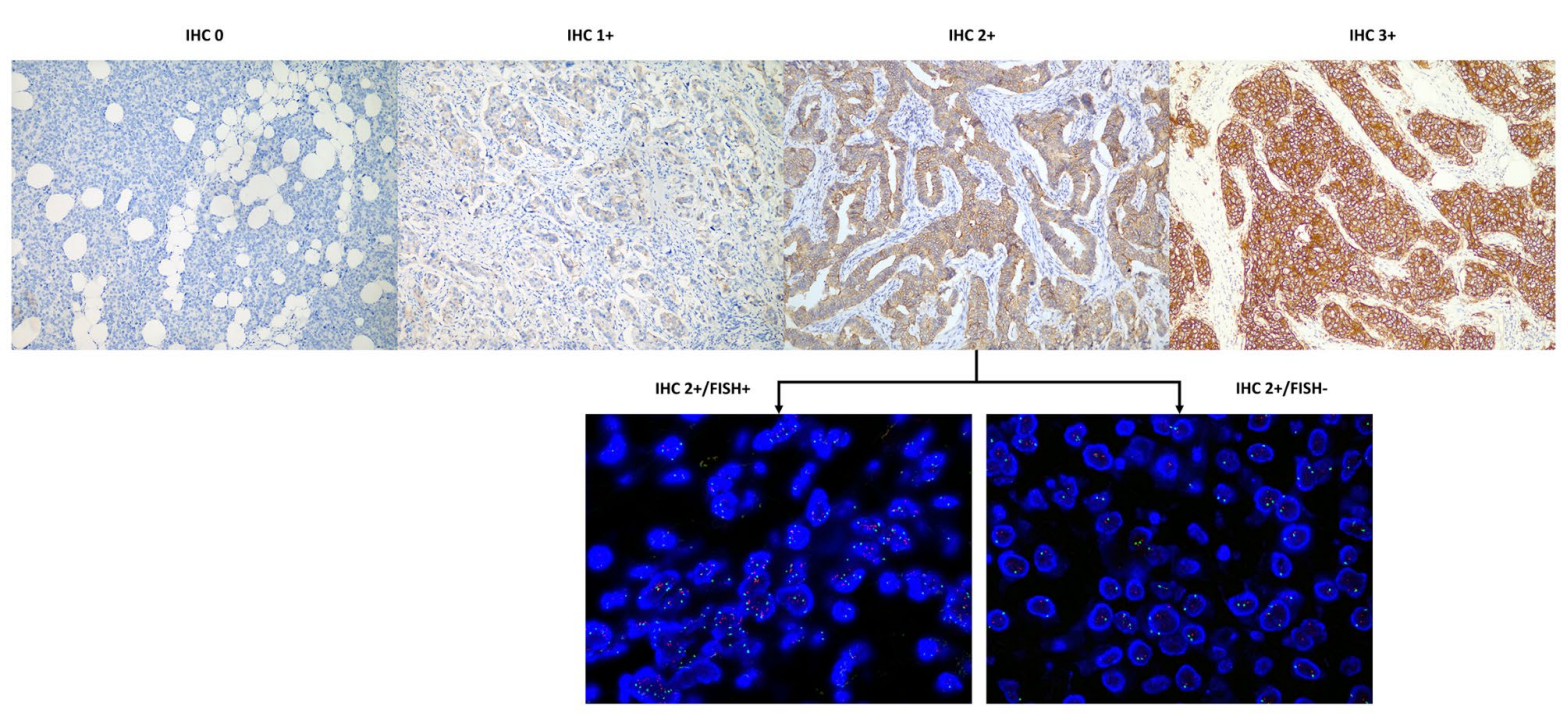
HER2 immunochemistry and fluorescence in situ hybridization showing representative cases for scoring

Overall, 53 (21.5%) samples of recurrent tumors were HER2-zero, 127 (51.4%) samples were HER2-low, and 67 (27.1%) samples were HER2-positive (Fig. 1). There was no significant difference in terms of age at recurrence (P = 0.268), BCFI (P = 0.217), site of biopsy or resection (P = 0.973) or Ki-67 index (P = 0.267) among the three HER2 groups. When categorized by the type of relapse, HER2-zero, HER2-low, and HER2-positive tumors represented 21.3%, 53.9%, and 24.7% of patients with LRR and 18.2%, 56.1%, and 25.8% of patients with CBC, respectively. Among patients with DM, 23.9% were HER2-zero, 45.7% were HER2-low, and 30.4% were HER2-positive (P = 0.703). When further using HR status to group breast cancer into subtypes, the HER2-zero, HER2-low, and HER2-positive subtypes comprised 17.0% (23), 68.1% (92), and 14.8% (20) of HR-positive recurrent tumors, respectively. However, the above three HER2 subtypes accounted for 26.8% (30), 31.3% (35), and 42.0% (47) of HR-negative tumors, respectively (P < 0.001, Fig. 1).

### Survival outcomes based on HER2 status at the site of relapse

**Fig. 2:**
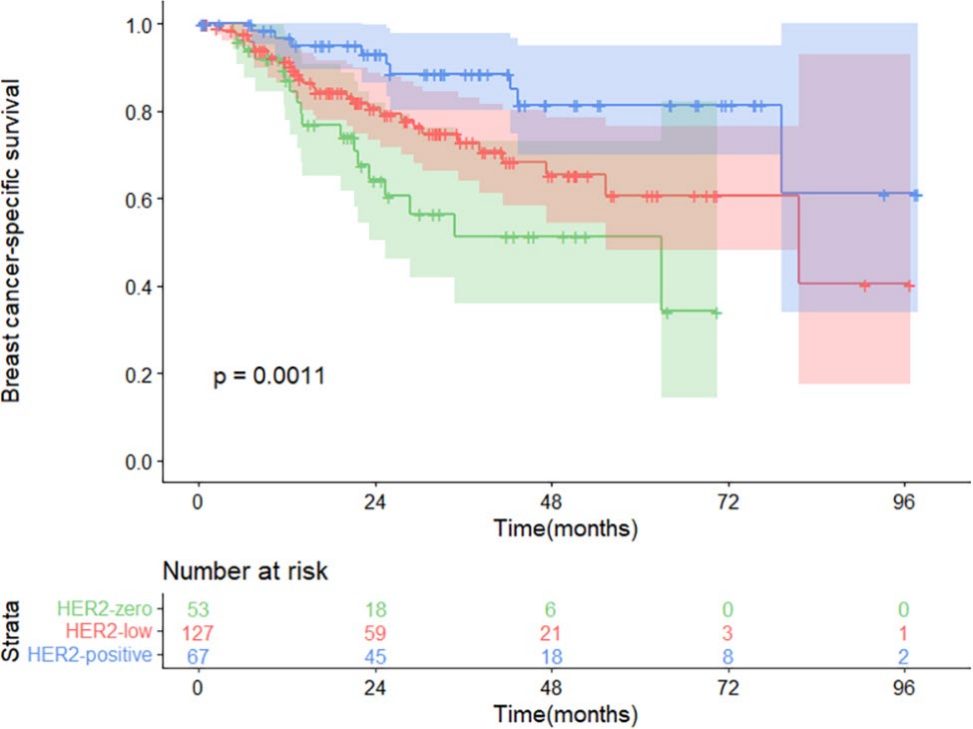
Kaplan-Meier survival analysis Comparison of Breast cancer-specific survival among three HER2 groups

The median follow-up time after disease recurrence was 23.3 (range, 0.5 to 97.9) months. A total of 57 deaths from breast cancer were observed in the whole population, and there were 18 deaths (34.0%), 30 deaths (23.6%), and 9 deaths (13.4%) in patients with recurrent tumors of the three HER2 subtypes. A significant difference in BCSS was observed when patients grouped by the HER2 status of recurrent lesions (P = 0.0011, Fig. 2). Univariate Cox analysis demonstrated that HER2-positive patients had better BCSS than HER2-low patients (hazard ratio = 0.422, 95% confidence interval [CI] = 0.199–0.892, P = 0.024), while the survival difference between the HER2-zero and HER2-low groups was only marginally significant (HR = 1.797, 95% CI = 0.997–3.239, P = 0.051).

**Fig. 3:**
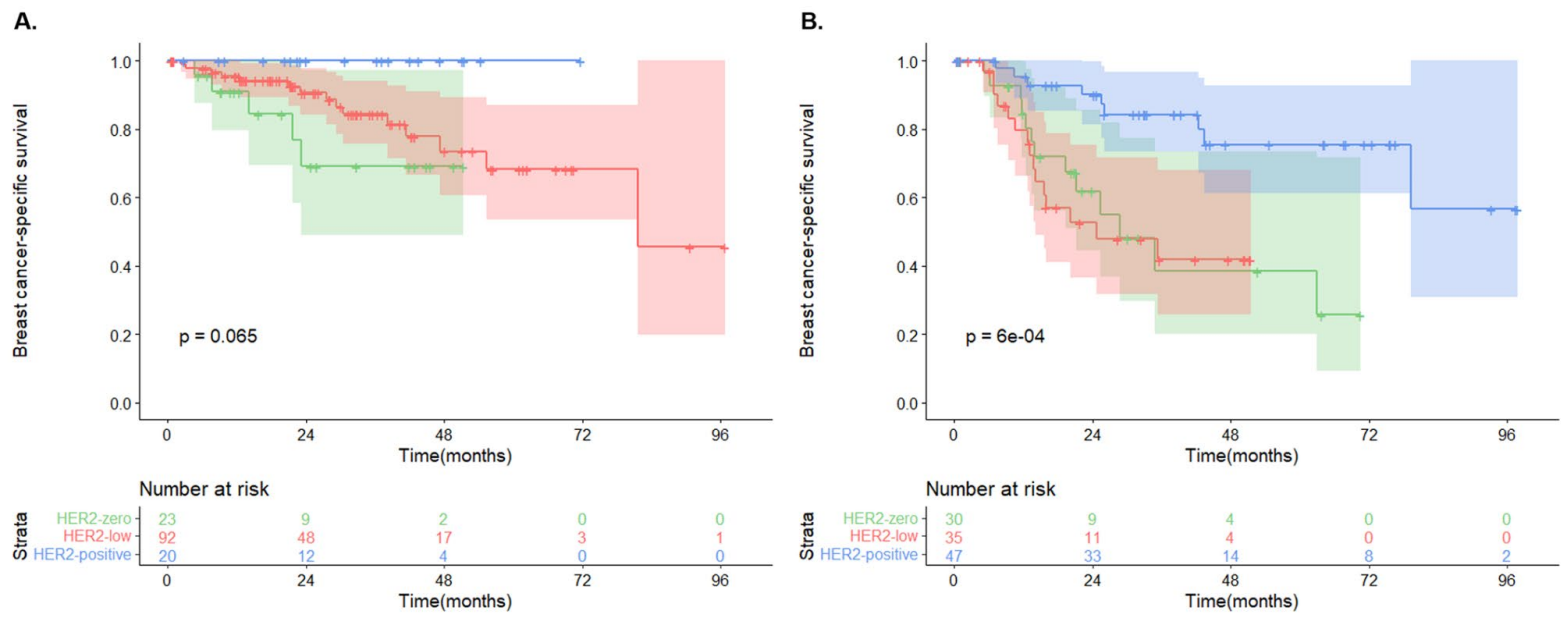
Kaplan-Meier survival analysis according to HR status Comparison of post-recurrence breast cancer-specific survival among three HER2 groups in HR-positive (A), and HR-negative (B) patients

**Fig. 4:**
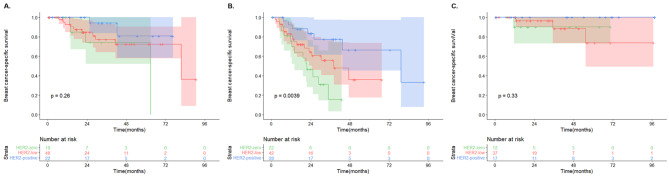
Kaplan-Meier survival analysis according to the type of relapse Comparison of post-recurrence breast cancer-specific survival among three HER2 groups in patients with LRR (A), DM (B), and CBC (C)

When the patients were further categorized by HR status, the survival difference among HER2 groups was only seen in those with HR-negative recurrent lesions (P = 0.0006) but not in those with HR-positive recurrent lesions (P = 0.065, Fig. 3). Moreover, there was a significant survival difference among HER2 groups in patients who had distant metastasis (P = 0.0039) but not in patients who had LRR (P = 0.26) or CBC (P = 0.33, Fig. 4).

Patients with HER2-positive recurrent tumors were then excluded from the analysis. Overall, HER2-low breast cancer tended to present a survival advantage (P = 0.053). When patients were stratified by HR status or type of relapse, no subgroup showed a significant survival difference between the HER2-negative and HER2-low cohorts (Figure S2).

### Exploratory analysis of HER2 status evolution and its impact on survival

**Fig. 5:**
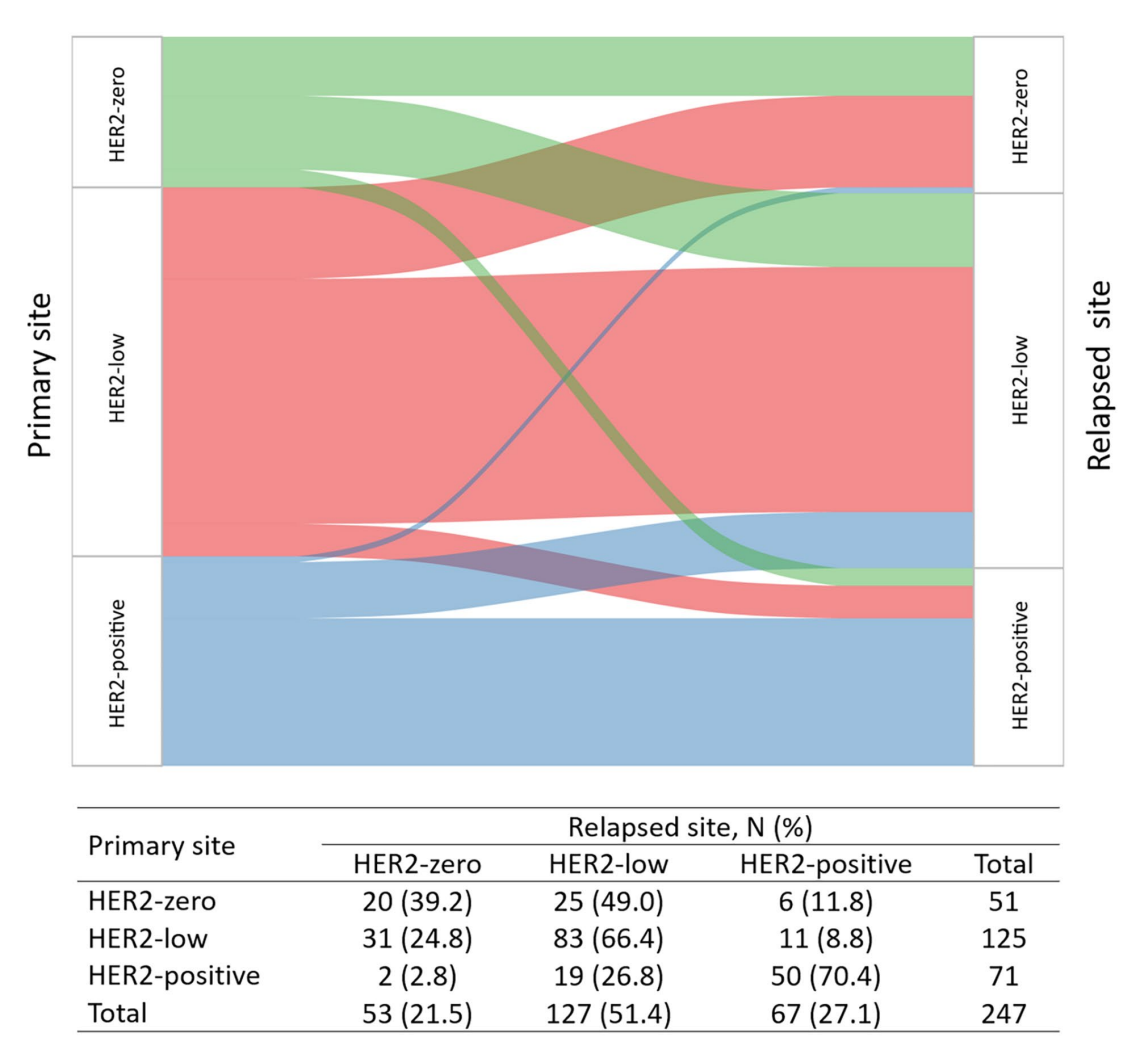
Evolution of HER2 status

The evolution of HER2 status between paired samples at diagnoses and recurrence is shown in Fig. 5. The overall rate of HER2 discordance was 38.1% (94 out of 247). After disease recurrence, 20 out of 51 (39.2%) patients with HER2-zero primary samples and 19 out of 71 (26.8%) patients with HER2-positive primary breast cancer samples showed a shift to HER2-low status. Moreover, 83 out of 125 (66.4%) HER2-low tumors remained the same status.

The rate of HER2 conversion was 40.0% in the HR-positive cohort and 35.7% in the HR-negative breast cancer cohort (P = 0.697, Figure S3). When grouped by the type of recurrence, 27.0% of patients with LRR and 30.4% of patients with DM had a shift in HER2 status, while 42 out of 66 (63.6%) patients with contralateral breast cancer had change in HER2 status (P < 0.001, Table S1, Figure S4).

Clinical outcomes were then evaluated according to the dynamic HER2 status during disease progression. The shift in HER2 status between primary and recurrent tumors did not affect BCSS (P = 0.61, Figure S5). Subgroup analysis demonstrated that only in cases of LRR did patients with concordant HER2 status have better survival than those who exhibited a change in HER2 status (P = 0.016).

## Discussion

In the current study, we described the distribution spectrum of HER2 expression in 247 patients with recurrent breast cancer. HER2-low breast cancer comprised 51.4% of the recurrent lesions, with a higher frequency in the HR-positive cohorts. Patients with HER2-low relapsed breast cancer had better survival than those with HER2-zero disease. Moreover, patients with HER2-positive recurrent tumors had the best clinical outcome after disease recurrence. During disease progression, the discordance rate of HER2 status was 38.1%, and 17.8% of tumors shifted to HER2-low status, and these patients may need more aggressive treatment after disease relapse.

The new term “HER2-low” brings a paradigm shift in the traditional binary categorization of HER2 status, which further dissects the heterogeneity within breast cancer [7]. In this work, 51.4% of recurrent lesions were HER2-low tumors, consistent with previous evidence. In early disease settings, recent research reported that HER2-low patients had a heavier tumor burden and lower Ki-67 index than HER2-zero patients [6, 8]. Genetic characteristics, including TP53, PIK3CA, ESR1, and PAM50 intrinsic subtypes, are also altered between HER2-zero and HER2-low breast cancer [6–8]. However, the NSABP B-47/NRG Oncology trial showed that additional trastuzumab based on adjuvant therapy cannot bring survival benefit for HER2-low patients, despite a certain amount of targetable HER2 [9]. Thus, in clinical practice, HER2-low early-stage breast cancer is currently treated as the HER2-negative entity. Our study focused on the advanced disease setting, and we found that a higher proportion of HER2-low cancer was seen in the HR-positive cohort. A similar scenario was reported by Miglitta et al. (53.8% vs. 36.2%) [23]. A possible reason is that the crosstalk between growth factors and estrogen signaling pathways could not only promote cell growth and proliferation but could also enhance resistance to endocrine therapy [24]. The interdependence between two pathways also has important implications for treatment, as blocking one pathway can lead to the upregulation and activation of another, leading to drug resistance [25, 26].

Overall, there is a lack of prospective data to support the prognostic value of the HER2-low subtype [6, 7, 10]. Since the benefit from novel ADCs for the HER2-low entity was mainly observed in patients with advanced disease, our study demonstrated the clinical outcomes of recurrent breast cancer using a three-group HER2 classification system. We found that HER2-positive breast cancer patients had the best survival after disease recurrence. This echoed the results from BCIRG 005/006, which showed better overall survival for HER2-positive patients treated with trastuzumab than traditional HER2-negative groups [27, 28]. Meanwhile, Grinda et al. demonstrated that the overall survival of HER2 + metastatic has been dramatically improved in the past decades, which may attribute to the advances in precise HER2-directed therapies. Moreover, when separated by the type of first recurrence, we noticed that only in patients with distant recurrence, was the survival among the three HER2 groups differ significantly. The plausible reason was that therapeutic strategies for LRR or CBC were with curative intention, whereas the therapeutic goals of metastatic breast cancer were improving the length and quality of life, which can better reflect the influence of tumor biology on prognosis. The specific anti-HER2 agent administered could affect these results. This could also to some extent indicate that HER2-low patients require more aggressive treatment after disease recurrence. Moreover, we found that the survival difference between patients with HER2-low and HER2-zero recurrent disease was not significant, but the HER2-low cohort tended to have better survival. The survival observations for these patients are still in conflict those in studies in early-stage breast cancer. The results from The Cancer Genome Atlas as well as the cBioPortal for Cancer Genomics demonstrated that the HER2-low subgroup and HER2-zero subgroup had no significant survival difference [7, 8]. However, a pooled analysis of large cohorts in clinical trials showed that patients with HER2-low tumors had significantly better survival than those with HER2-zero tumors [6]. These findings suggest once more that current data are insufficient to define the HER2-low subgroup as an individual breast cancer subtype with a distinct prognosis. Further studies with larger populations and longer follow-up are eagerly awaited, and these studies should distinguish between the luminal and triple-negative subgroups to eliminate the impact of endocrine treatment.

The rate of HER2 status discordance between breast cancer tumors at first diagnosis and recurrence was 38.1% in this work, similar to the results from Miglietta et al. [23]. When only considering the dichotomous classification of HER2 status (negative vs. positive), the majority of patients with discordance (22.0%) were excluded, and our result was consistent with previous studies (a 10–15% discordance rate) [29, 30]. The inconsistency may be attributed to intratumor heterogeneity of the primary lesion [31], the stochastic changes induced by bidirectional interactions between cells and the extracellular matrix [32], or the emergence of new subclones under selective pressure, including treatment [33]. Moreover, we noticed that a total of 44 (17.8%) cases shifted to HER2-low status after disease recurrence. These included 49.0% of primary HER2-zero tumors and 26.8% of primary HER2-positive tumors. Recent studies have suggested that novel ADCs harboring a higher drug-to-antibody ratio (DAR) and/or cleavable linkers may facilitate the killing not only of bystander HER2-positive cells but also of neighboring non–antigen-expressing cells, potentially overcoming the intratumor heterogeneity in HER2 expression. Trastuzumab deruxtecan (T-DXd; formerly DS-8201a), composed of trastuzumab and a topoisomerase I inhibitor, could yield about an ORR of 37% with a median duration of response (DoR) of 10.4 months in 54 HER2-low breast cancer patients with disease refractory to standard therapies [16]. Similar results were observed with trastuzumab duocarmazine (SYD985), another HER2-targeting ADC with a cleavable linker-duocarmycin payload. Based on the above, phase III clinical trials are ongoing to compare the novel ADCs with chemotherapy of the physician’s choice in HER2-low advanced disease [17, 18]. Our results indicated that nearly one-fifth of patients might potentially benefit from the revolutionized anti-HER2 approach after disease relapse, especially those with primary HER2-zero tumors. From another point of view, HER2 conversion during disease progression encouraged the pathological reassessment of local-regional or metastatic recurrence to provide rational evidence for subsequent treatment.

To our knowledge, after the introduction of the HER2-low breast cancer classification, there has been limited research focused on the prognosis of recurrent breast cancer or HER2 status evolution during disease progression. There are also potential limitations to our work. First, the small sample size is a shortcoming that cannot be ignored. Second, selection bias was inevitable since we only included patients with paired samples for the primary and recurrent tumors. Moreover, patients were retrospectively collected over a long period of nearly ten years, and the treatment paradigm had changed greatly, which may have an influence on the clinical outcomes. Thus, when analyzing the survival of patients, the treatment strategies after disease relapse should be further taken into account. Last but not least, there was a lack of data based on gene expression, and such data may provide a clearer picture of disease heterogeneity and warrant exploration in the future.

In conclusion, HER2-low tumors accounted for approximately 50% of recurrent and metastatic breast cancer and comprised more of the HR-positive subtype. HER2-positive relapsed breast cancer was associated with the best clinical outcome after disease recurrence, whereas the survival difference was only marginally better in the HER2-low group than in the HER2-zero group. HER2 status was not stable during disease progression, with approximately one-fifth of patients exhibiting a shift to HER2-low status. Those patients warrant more aggressive treatment after disease relapse, which needs further research.

## Electronic supplementary material

Below is the link to the electronic supplementary material.

Supplementary Material 1

## Data Availability

The datasets generated during and/or analyzed during the current study are available from the corresponding author on reasonable request at http://bcdb.mdt.team:8080.

## Abbreviations

HER2: human epidermal growth factor receptor-2
IHC: immunohistochemistry
FISH: fluorescence in situ hybridization
BCSS: breast cancer-specific survival
HR: hormone receptor
ASCO/CAP: American Society of Clinical Oncology/College of American Pathologists
ADC: anti-drug conjugate
ORR: objective response rate
LRR: local-regional recurrence
DM: distant metastasis
CBC: contralateral breast cancer
CI: confidence interval

## References

[1] Slamon DJ, Clark GM, Wong SG, Levin WJ, Ullrich A, McGuire WL (1987). Human breast cancer: correlation of relapse and survival with amplification of the HER-2/neu oncogene. Science.

[2] Seshadri R, Firgaira FA, Horsfall DJ, McCaul K, Setlur V, Kitchen P (1993). Clinical significance of HER-2/neu oncogene amplification in primary breast cancer. The South australian breast Cancer Study Group. J Clin Oncol.

[3] Loibl S, Gianni L (2017). HER2-positive breast cancer. Lancet.

[4] Wolff AC, Hammond MEH, Allison KH, Harvey BE, Mangu PB, Bartlett JMS, Bilous M, Ellis IO, Fitzgibbons P, Hanna W, Jenkins RB, Press MF, Spears PA, Vance GH, Viale G, McShane LM, Dowsett M (2018). Human epidermal growth factor receptor 2 testing in breast Cancer: American Society of Clinical Oncology/College of American Pathologists Clinical Practice Guideline focused Update. J Clin Oncol.

[5] Tarantino P, Hamilton E, Tolaney SM, Cortes J, Morganti S, Ferraro E, Marra A, Viale G, Trapani D, Cardoso F, Penault-Llorca F, Viale G, Andrè F, Curigliano G (2020). HER2-Low breast Cancer: pathological and clinical Landscape. J Clin Oncol.

[6] Denkert C, Seither F, Schneeweiss A, Link T, Blohmer J-U, Just M, Wimberger P, Forberger A, Tesch H, Jackisch C, Schmatloch S, Reinisch M, Solomayer EF, Schmitt WD, Hanusch C, Fasching PA, Lübbe K, Solbach C, Huober J, Rhiem K, Marmé F, Reimer T, Schmidt M, Sinn BV, Janni W, Stickeler E, Michel L, Stötzer O, Hahnen E, Furlanetto J, Seiler S, Nekljudova V, Untch M, Loibl S (2021). Clinical and molecular characteristics of HER2-low-positive breast cancer: pooled analysis of individual patient data from four prospective, neoadjuvant clinical trials. Lancet Oncol.

[7] Agostinetto E, Rediti M, Fimereli D, Debien V, Piccart M, Aftimos P, Sotiriou C, de Azambuja EJC. (2021) HER2-Low Breast Cancer: Molecular Characteristics and Prognosis. 13 (11):2824 10.3390/cancers13112824 PMC8201345 34198891

[8] Schettini F, Chic N, Brasó-Maristany F, Paré L, Pascual T, Conte B, Martínez-Sáez O, Adamo B, Vidal M, Barnadas E, Fernández-Martinez A, González-Farre B, Sanfeliu E, Cejalvo JM, Perrone G, Sabarese G, Zalfa F, Peg V, Fasani R, Villagrasa P, Gavilá J, Barrios CH, Lluch A, Martín M, Locci M, De Placido S, Prat A (2021). Clinical, pathological, and PAM50 gene expression features of HER2-low breast cancer. NPJ Breast Cancer.

[9] Fehrenbacher L, Cecchini RS, Geyer CE, Rastogi P, Costantino JP, Atkins JN, Crown JP, Polikoff J, Boileau J-F, Provencher L, Stokoe C, Moore TD, Robidoux A, Flynn PJ, Borges VF, Albain KS, Swain SM, Paik S, Mamounas EP, Wolmark N (2020). NSABP B-47/NRG oncology phase III Randomized Trial comparing adjuvant chemotherapy with or without Trastuzumab in high-risk invasive breast Cancer negative for HER2 by FISH and with IHC 1 + or 2. J Clin Oncol.

[10] Rossi V, Sarotto I, Maggiorotto F, Berchialla P, Kubatzki F, Tomasi N, Redana S, Martinello R, Valabrega G, Aglietta M, Ponzone R, Montemurro F (2012). Moderate immunohistochemical expression of HER-2 (2+) without HER-2 gene amplification is a negative prognostic factor in early breast cancer. Oncologist.

[11] Eggemann H, Ignatov T, Burger E, Kantelhardt EJ, Fettke F, Thomssen C, Costa SD, Ignatov A (2015). Moderate HER2 expression as a prognostic factor in hormone receptor positive breast cancer. Endocr Relat Cancer.

[12] van der Lee MMC, Groothuis PG, Ubink R, van der Vleuten MAJ, van Achterberg TA, Loosveld EM, Damming D, Jacobs DCH, Rouwette M, Egging DF, van den Dobbelsteen D, Beusker PH, Goedings P, Verheijden GFM, Lemmens JM, Timmers M, Dokter WHA (2015). The Preclinical Profile of the Duocarmycin-Based HER2-Targeting ADC SYD985 predicts for Clinical Benefit in Low HER2-Expressing breast cancers. Mol Cancer Ther.

[13] Takegawa N, Tsurutani J, Kawakami H, Yonesaka K, Kato R, Haratani K, Hayashi H, Takeda M, Nonagase Y, Maenishi O, Nakagawa K (2019). [fam-] trastuzumab deruxtecan, antitumor activity is dependent on HER2 expression level rather than on HER2 amplification. Int J Cancer.

[14] Skidmore L, Sakamuri S, Knudsen NA, Hewet AG, Milutinovic S, Barkho W, Biroc SL, Kirtley J, Marsden R, Storey K, Lopez I, Yu W, Fang S-Y, Yao S, Gu Y, Tian F (2020). ARX788, a site-specific Anti-HER2 antibody-drug Conjugate, demonstrates potent and selective activity in HER2-low and T-DM1-resistant breast and gastric cancers. Mol Cancer Ther.

[15] Banerji U, van Herpen CML, Saura C, Thistlethwaite F, Lord S, Moreno V, Macpherson IR, Boni V, Rolfo C, de Vries EGE, Rottey S, Geenen J, Eskens F, Gil-Martin M, Mommers EC, Koper NP, Aftimos P (2019). Trastuzumab duocarmazine in locally advanced and metastatic solid tumours and HER2-expressing breast cancer: a phase 1 dose-escalation and dose-expansion study. Lancet Oncol.

[16] Modi S, Park H, Murthy RK, Iwata H, Tamura K, Tsurutani J, Moreno-Aspitia A, Doi T, Sagara Y, Redfern C, Krop IE, Lee C, Fujisaki Y, Sugihara M, Zhang L, Shahidi J, Takahashi S (2020). Antitumor Activity and Safety of Trastuzumab Deruxtecan in patients with HER2-Low-expressing advanced breast Cancer: results from a phase ib study. J Clin Oncol.

[17] Bardia A, Barrios C, Dent R, Hu X, O’Shaughnessy J, Yonemori K, Darilay A, Boston S, Liu Y, Patel G. Trastuzumab deruxtecan (T-DXd; DS-8201) vs investigator’s choice of chemotherapy in patients with hormone receptor-positive (HR+), HER2 low metastatic breast cancer whose disease has progressed on endocrine therapy in the metastatic setting: A randomized, global phase 3 trial (DESTINY-Breast06). In: CANCER RESEARCH, 2021. vol 4. AMER ASSOC CANCER RESEARCH 615 CHESTNUT ST, 17TH FLOOR, PHILADELPHIA, PA &#8230

[18] Modi S, Ohtani S, Lee C, Wang Y, Saxena K, Cameron DA. Abstract OT1-07-02: a phase 3, multicenter, randomized, open-label trial of [fam-] trastuzumab deruxtecan (T-DXd; DS-8201a) vs investigator’s choice in HER2-low breast cancer (DESTINY-Breast04). AACR; 2020.

[19] Tolaney SM, Garrett-Mayer E, White J, Blinder VS, Foster JC, Amiri-Kordestani L, Hwang ES, Bliss JM, Rakovitch E, Perlmutter J, Spears PA, Frank E, Tung NM, Elias AD, Cameron D, Denduluri N, Best AF, DiLeo A, Baizer L, Butler LP, Schwartz E, Winer EP, Korde LA (2021). Updated standardized definitions for efficacy end points (STEEP) in adjuvant breast Cancer clinical trials: STEEP Version 2.0. J Clin Oncol:JCO2003613.

[20] Tong Y, Chen X, Fei X, Lin L, Wu J, Huang O, He J, Zhu L, Chen W, Li Y, Shen K. Can breast cancer patients with HER2 dual-equivocal tumours be managed as HER2-negative disease? Eur J Cancer. 2018;89. 10.1016/j.ejca.2017.10.033. 10.1016/j.ejca.2017.10.033 29223481

[21] Wolff AC, Hammond MEH, Schwartz JN, Hagerty KL, Allred DC, Cote RJ, Dowsett M, Fitzgibbons PL, Hanna WM, Langer A, McShane LM, Paik S, Pegram MD, Perez EA, Press MF, Rhodes A, Sturgeon C, Taube SE, Tubbs R, Vance GH, van de Vijver M, Wheeler TM, Hayes DF (2007). American Society of Clinical Oncology/College of American Pathologists guideline recommendations for human epidermal growth factor receptor 2 testing in breast cancer. J Clin Oncol.

[22] Wolff AC, Hammond MEH, Hicks DG, Dowsett M, McShane LM, Allison KH, Allred DC, Bartlett JMS, Bilous M, Fitzgibbons P, Hanna W, Jenkins RB, Mangu PB, Paik S, Perez EA, Press MF, Spears PA, Vance GH, Viale G, Hayes DF (2013). Recommendations for human epidermal growth factor receptor 2 testing in breast cancer: american society of clinical Oncology/College of american pathologists clinical practice guideline update. J Clin Oncol.

[23] Miglietta F, Griguolo G, Bottosso M, Giarratano T, Lo Mele M, Fassan M, Cacciatore M, Genovesi E, De Bartolo D, Vernaci GJNbc. (2021) Evolution of HER2-low expression from primary to recurrent breast cancer. 7 (1):1–8 10.1038/s41523-021-00343-4 PMC8511010 34642348

[24] Konecny G, Pauletti G, Pegram M, Untch M, Dandekar S, Aguilar Z, Wilson C, Rong H-M, Bauerfeind I, Felber M, Wang H-J, Beryt M, Seshadri R, Hepp H, Slamon DJ (2003). Quantitative association between HER-2/neu and steroid hormone receptors in hormone receptor-positive primary breast cancer. J Natl Cancer Inst.

[25] Arpino G, Wiechmann L, Osborne CK, Schiff R (2008). Crosstalk between the estrogen receptor and the HER tyrosine kinase receptor family: molecular mechanism and clinical implications for endocrine therapy resistance. Endocr Rev.

[26] Sudhan DR, Schwarz LJ, Guerrero-Zotano A, Formisano L, Nixon MJ, Croessmann S, González Ericsson PI, Sanders M, Balko JM, Avogadri-Connors F, Cutler RE, Lalani AS, Bryce R, Auerbach A, Arteaga CL (2019). Extended adjuvant therapy with Neratinib Plus Fulvestrant Blocks ER/HER2 crosstalk and maintains complete responses of ER/HER2 breast cancers: implications to the ExteNET Trial. Clin Cancer Res.

[27] Mackey JR, Pieńkowski T, Crown J, Sadeghi S, Martin M, Chan A, Saleh M, Sehdev S, Provencher L, Semiglazov V, Press MF, Sauter G, Lindsay M, Houé V, Buyse M, Drevot P, Hitier S, Bensfia S, Eiermann W (2016). Long-term outcomes after adjuvant treatment of sequential versus combination docetaxel with doxorubicin and cyclophosphamide in node-positive breast cancer: BCIRG-005 randomized trial. Ann Oncol.

[28] Slamon D, Eiermann W, Robert N, Giermek J, Martin M, Jasiowka M, Mackey J, Chan A, Liu M, Pinter T. Abstract S5-04: ten year follow-up of BCIRG-006 comparing doxorubicin plus cyclophosphamide followed by docetaxel (AC→ T) with doxorubicin plus cyclophosphamide followed by docetaxel and trastuzumab (AC→ TH) with docetaxel, carboplatin and trastuzumab (TCH) in HER2 + early breast cancer. AACR; 2016.

[29] Yeung C, Hilton J, Clemons M, Mazzarello S, Hutton B, Haggar F, Addison CL, Kuchuk I, Zhu X, Gelmon K, Arnaout A (2016). Estrogen, progesterone, and HER2/neu receptor discordance between primary and metastatic breast tumours-a review. Cancer Metastasis Rev.

[30] Walter V, Fischer C, Deutsch TM, Ersing C, Nees J, Schütz F, Fremd C, Grischke E-M, Sinn P, Brucker SY, Schneeweiss A, Hartkopf AD, Wallwiener M (2020). Estrogen, progesterone, and human epidermal growth factor receptor 2 discordance between primary and metastatic breast cancer. Breast Cancer Res Treat.

[31] McGranahan N, Swanton C (2017). Clonal heterogeneity and Tumor Evolution: past, Present, and the future. Cell.

[32] Lüönd F, Tiede S, Christofori G (2021). Breast cancer as an example of tumour heterogeneity and tumour cell plasticity during malignant progression. Br J Cancer.

[33] Kan S, Koido S, Okamoto M, Hayashi K, Ito M, Kamata Y, Komita H, Ishidao T, Nagasaki E, Homma S (2015). Gemcitabine treatment enhances HER2 expression in low HER2-expressing breast cancer cells and enhances the antitumor effects of trastuzumab emtansine. Oncol Rep.

[34] Modi Shanu, Jacot William, Yamashita Toshinari, Sohn Joohyuk, Vidal Maria, Tokunaga Eriko, Tsurutani Junji, Ueno Naoto T., Chae Yee Soo, Lee Keun Seok, Niikura Naoki, Park Yeon Hee, Wang Xiaojia, Xu Binghe, Gambhire Dhiraj, Yung Lotus, Meinhardt Gerold, Wang Yibin, Harbeck Nadia, Cameron David A. (2022). Trastuzumab deruxtecan (T-DXd) versus treatment of physician’s choice (TPC) in patients (pts) with HER2-low unresectable and/or metastatic breast cancer (mBC): Results of DESTINY-Breast04, a randomized, phase 3 study.. Journal of Clinical Oncology.

