# Supplementary material for: Distribution, dynamic evolution, and clinical outcomes of patients with advanced breast cancer according to HER2 expression
Source: BMC Cancer. 2023 Feb 21;23:173. doi: 10.1186/s12885-023-10634-7 (PMC9942407; doi:10.1186/s12885-023-10634-7)
Supplement: Supplementary file 1 — Supplementary Material 1 [file 12885_2023_10634_MOESM1_ESM.doc]

**Table S1** HER2 evolution according to HR status and site of recurrence

|  |  | HER2-zero | HER2-low | HER2-positive | Total |
| --- | --- | --- | --- | --- | --- |
| HR-positive | HER2-zero | 9 (31.0) | 18 (62.1) | 2 (6.9) | 29 |
|  | HER2-low | 13 (16.9) | 59 (76.6) | 5 (6.5) | 77 |
|  | HER2-positive | 1 (3.4) | 15 (51.7) | 13 (44.8) | 29 |
|  | Total | 23 (17.0) | 92 (68.1) | 20 (14.8) | 135 |
| HR-negative | HER2-zero | 11 (50.0) | 7 (31.8) | 4 (18.2) | 22 |
|  | HER2-low | 18 (37.5) | 24 (50.0) | 6 (12.5) | 48 |
|  | HER2-positive | 1 (2.4) | 4 (9.5) | 37 (88.1) | 42 |
|  | Total | 30 (26.8) | 35 (31.3) | 47 (42.0) | 112 |
| LRR | HER2-zero | 11 (55.0) | 9 (45.0) | 0 (0.0) | 20 |
|  | HER2-low | 8 (17.4) | 35 (76.1) | 3 (6.5) | 46 |
|  | HER2-positive | 0 (0.0) | 4 (17.4) | 19 (82.6) | 23 |
|  | Total | 19 (21.3) | 48 (53.9) | 22 (24.7) | 89 |
| DR | HER2-zero | 6 (42.9) | 7 (50.0) | 1 (7.1) | 14 |
|  | HER2-low | 15 (30.0) | 33 (66.0) | 2 (4.0) | 50 |
|  | HER2-positive | 1 (3.6) | 2 (7.1) | 25 (89.3) | 28 |
|  | Total | 22 (23.9) | 42 (45.7) | 28 (30.4) | 92 |
| CBC | HER2-zero | 3 (17.6) | 9 (52.9) | 5 (29.4) | 17 |
|  | HER2-low | 8 (27.6) | 15 (51.7) | 6 (20.7) | 29 |
|  | HER2-positive | 1 (5.0) | 13 (65.0) | 6 (30.0) | 20 |
|  | Total | 12 (18.2) | 37 (56.1) | 17 (25.8) | 66 |

**Figure S1** Flow chart


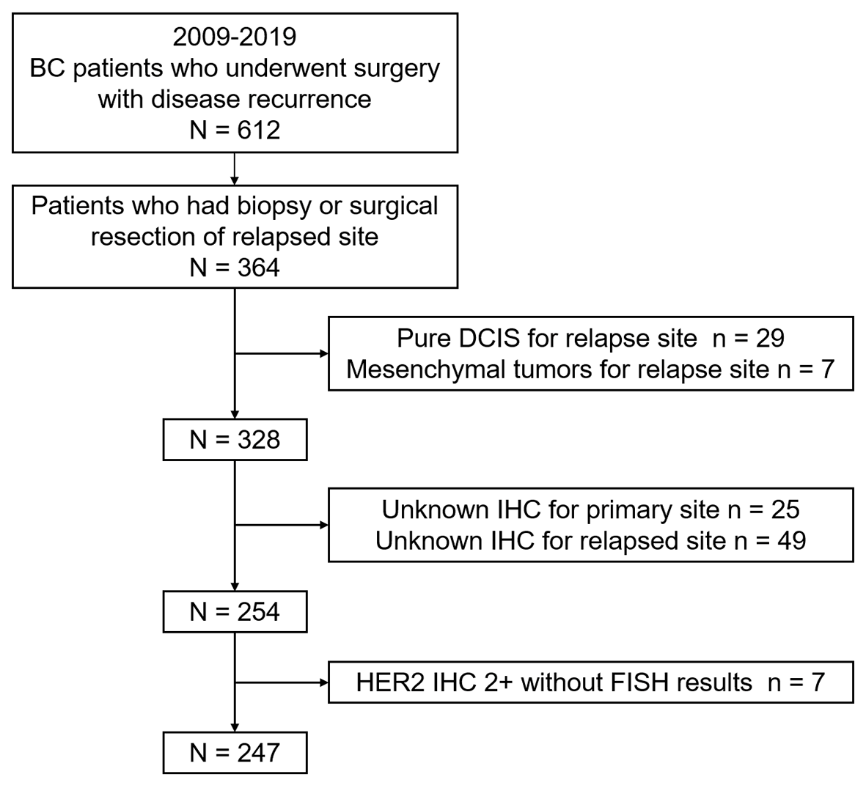


**Figure S2** Kaplan-Meier curves between HER2-zero and HER2-low cohorts


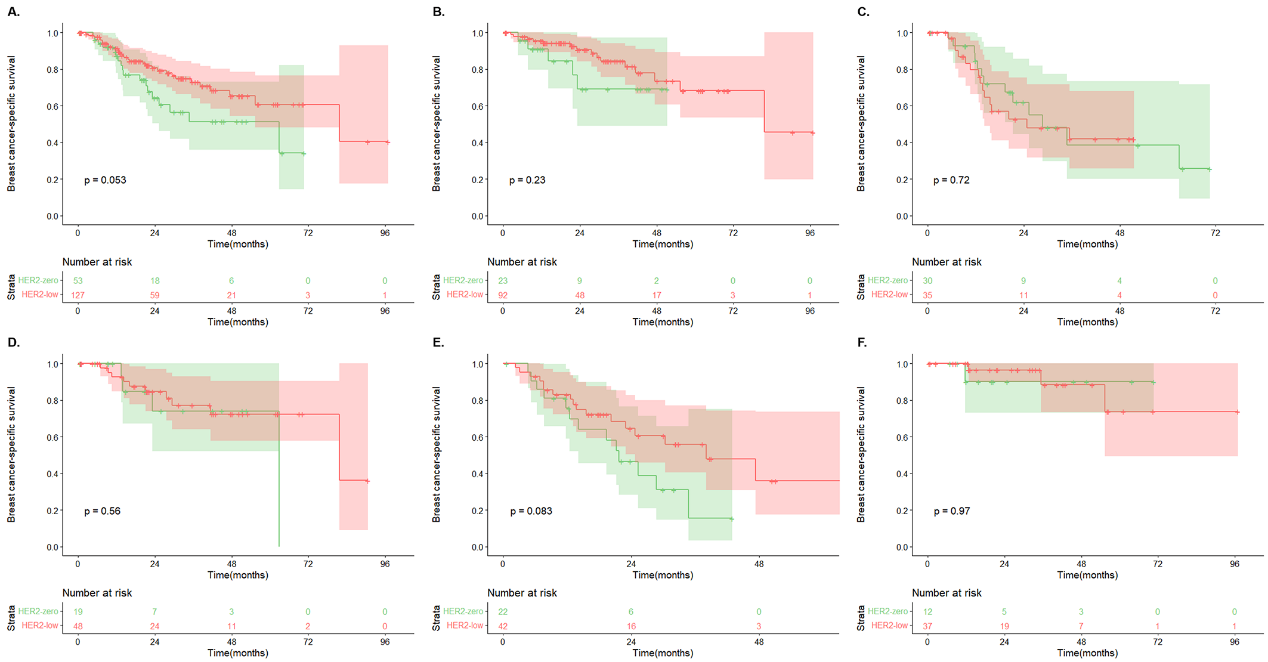


Patients with HER2-positive relapsed breast cancer were excluded from the analysis, and the clinical outcome were compared between HER2-zero and HER2-low cohorts in the rest of population (A), and in patients with HR+ relapsed breast cancer (B), HR- relapsed breast cancer (C), loco-regional recurrence (D), distant metastasis (E), and contralateral breast cancer.

**Figure S3** Evolution of HER2 status according to HR status


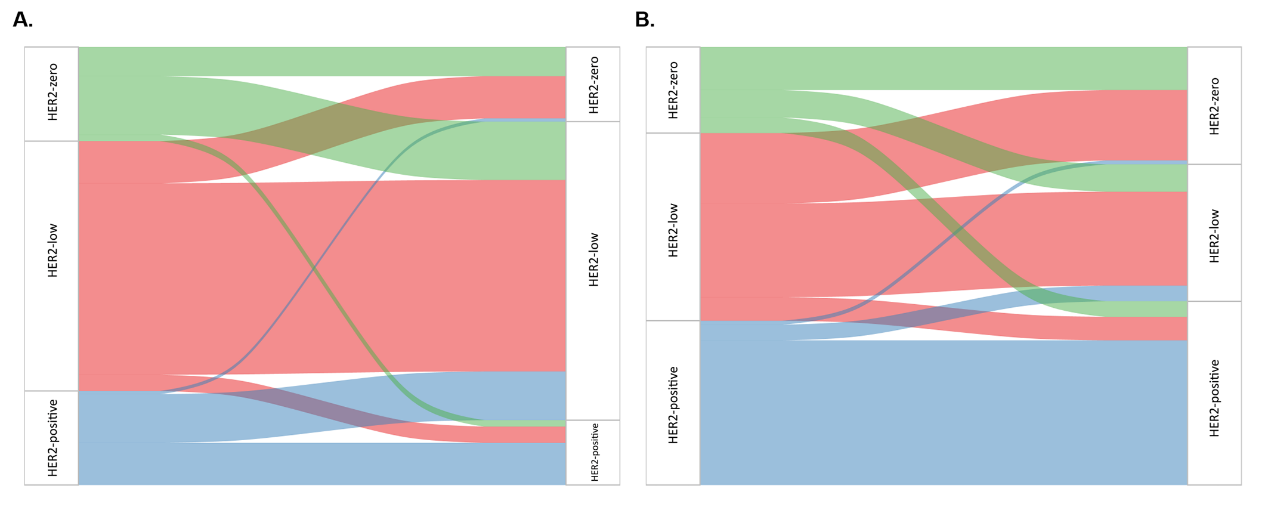


Evolution of HER2 status in HR-positive relapsed breast cancer (A), and HR-negative patients (B).

**Figure S4** Evolution of HER2 status according to type of first relapse


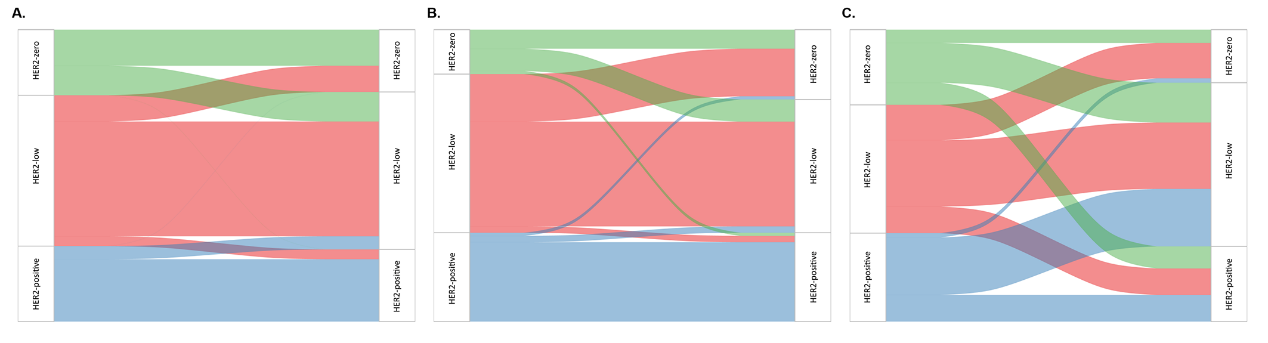


Evolution of HER2 status in patients with LRR (A), DM (B), and CBC (C).

**Figure S5** Kaplan-Meier curves according to conversion of HER2 status


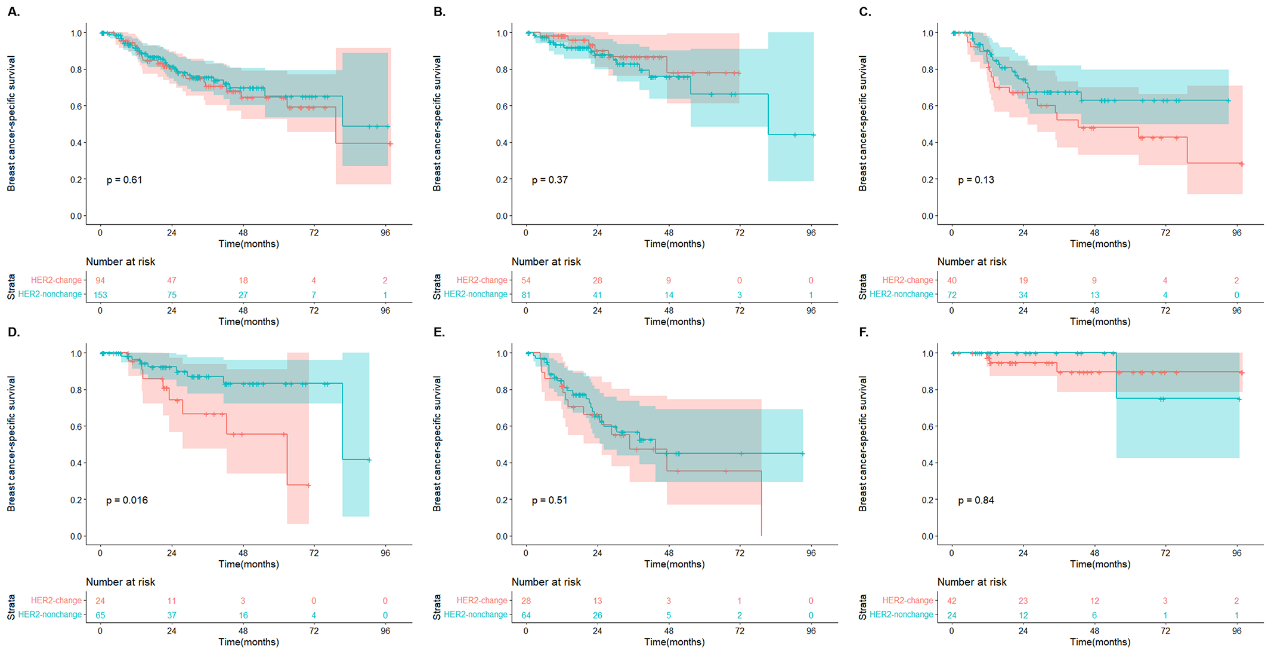


Kaplan-Meier curves of patients who underwent a change in HER2 status during disease progression or not in all patients (A), and in patients with HR+ relapsed breast cancer (B), HR- relapsed breast cancer (C), loco-regional recurrence (D), distant metastasis (E), and contralateral breast cancer (F).
